# Flavor Wheel Development from a Machine Learning Perspective

**DOI:** 10.3390/foods13244142

**Published:** 2024-12-20

**Authors:** Anggie V. Rodríguez-Mendoza, Santiago Arbeláez-Parra, Rafael Amaya-Gómez, Nicolas Ratkovich

**Affiliations:** 1 Department of Chemical & Food Engineering, Universidad de los Andes, Cra. 1E No. 19a-40, Bogotá D.C. 111711, Colombia; av.rodriguezm1@uniandes.edu.co (A.V.R.-M.); s.arbelaezp@uniandes.edu.co (S.A.-P.); 2 Department of Industrial Engineering, Universidad de los Andes, Cra. 1E No. 19a-40, Bogotá D.C. 111711, Colombia

**Keywords:** flavor wheel, machine learning, PCA, distilled spirit, chemical compounds

## Abstract

The intricate relationships between chemical compounds and sensory descriptors in distilled spirits have long intrigued distillers, sensory experts, and consumers alike. The importance and complexity of this relation affect the production, quality, and appreciation of spirits, and the success of a product. Because of that, profoundly investigating the different flavor and aroma combinations that the chemical compounds can give to a desired beverage takes an essential place in the industry. This study aims to study these relationships by employing machine learning techniques to analyze a comprehensive dataset with 3051 chemical compounds and their associated aroma descriptors for seven distilled spirit categories: Baijiu, cachaça, gin, mezcal, rum, tequila, and whisk(e)y. The study uses principal component analysis (PCA) to reduce the dimensionality of the dataset and a clustering machine learning model to identify distinct clusters of aroma descriptors associated with each beverage category. Based on these results, an aroma wheel that encapsulates the diverse olfactory landscapes of various distilled spirits was developed. This flavor wheel is a valuable tool for distillers, sensory experts, and consumers, providing a comprehensive reference for understanding and appreciating the complexities of distilled spirits.

## 1. Introduction

Spirits and alcoholic beverages encompass various elixirs that have been distilled and often aged to attain unique and complex flavor profiles. These beverages, ranging from cachaça and brandy to rum and cognac, are cherished not only for their alcoholic content but also for the richness and depth of their flavor notes and aromas. The sensory diversity of spirits originates from an intricate interplay of multiple factors [1,2]. The grains, fruits, or vegetables used in fermentation and distillation contribute fundamental nuances, while the aging process in oak casks adds additional layers of complexity. The duration of aging, climatic conditions, and the type of wood used in the cask construction significantly influence the final profile. These beverages contain a variety of compounds such as esters, aldehydes, and phenols, among others, which are fundamental to the flavor and aroma profiles, directly influencing consumer preferences depending on the distribution and traces [3,4]. For instance, esters typically enhance the flavor profile with fruity notes. Understanding these chemical components allows producers to refine their fermentation and distillation processes, optimizing flavor extraction and product quality.

Each alcoholic spirit possesses a distinctive sensory signature. From the smoky and earthy notes of single malt whisk(e)y to the subtle fruity and vanilla hints of brandy, these beverages celebrate sensory diversity [2,5,6]. Sensory descriptors provide a language for consumers to articulate their tasting experiences, enhancing their enjoyment and guiding their preferences. They serve as benchmarks, allowing for comparisons across products and helping producers craft unique flavor profiles. For instance, Sampaio et al. [7] described the production of a spirit from spent coffee grounds, identifying 17 volatiles and 12 sensory descriptors that allowed them to characterize the sensory profile. In the food industry, identifying and characterizing aromas and flavors are crucial to ensure product quality and enhance the culinary experience for consumers. This process, conducted by experts, highlights the impact of sensory experiences on our relationship with food and gastronomic culture. The precise identification and description of aromas and flavors are crucial in this process, allowing the creation and control of specific sensory profiles that underscore the quality and consistency of food products. The sensory evaluation of these flavor details is based on human perception, and it involves a mixture of qualitative and quantitative analysis that minimizes the subjectivity of the sensory evaluation. Each taster brings a unique set of sensory receptors shaped by genetics, experiences, and personal history [1,2,5]. This mosaic of influences converges to form a distinctive palate, where certain flavors and aromas vibrate more vividly while others may remain undisturbed. Within this context, the flavor wheel stands out as a vital resource. This systematic and standardized guide streamlines developing and evaluating new food products by enabling professionals to accurately identify and blend aromas to achieve specific sensory profiles [1,2,8].

Regarding flavor wheels’ development from sensory descriptors, varied research is available. For instance, Calvert et al. [9] evaluated the flavor wheel of cider using word sorting under a sensory descriptive analysis of 40 sensory professionals and multivariate statistical techniques. Also, Hamilton and Lahne [10] proposed an automated sensory analysis using natural language processing from a descriptive lexicon from two websites about whisk(e)y using web scraping. Interestingly, flavor wheels have also been contemplated in non-distilled beverages. Some cases include the work of Wang et al. [11], who developed an aroma wheel of Oolong tea under different storage environments. Yu et al. [12] studied the profile description of human milk. In this work, twelve sensory evaluators formed a sensory panel for evaluation using a lexicon screening regarding aroma, flavor, and mouth feeling. As noted, flavor wheels have traditionally been constructed using sensory descriptors and usually not chemical compounds. Some exceptions include the work of Ramírez et al. [13], where a botanical compatibility assessment was implemented in gin production. This work focused on how sensory and chemical compounds can be used to develop a more robust and general flavor wheel from a machine learning perspective.

Previous studies have meticulously laid the groundwork for this research, offering unique insights into utilizing innovative technologies in food and beverage evaluation. González-Viejo et al. [1] pioneered the development of a cost-effective electronic nose, revolutionizing the analysis of beer aromas and providing a reliable metric for quality assessment. Voss et al. [2] expanded this exploration by introducing an electronic nose designed to measure ethanol concentrations in alcoholic beverages, employing advanced mathematical techniques for precise quantification. These contributions collectively highlight the pivotal role of innovative technology in enhancing sensory analysis within the food and beverage industry. Building upon this foundation, subsequent studies have continued to push the boundaries of innovation in food evaluation methodologies. Hu et al. [5] introduced a versatile and cost-effective sensor propelled by machine learning capabilities, enabling the classification of diverse coffee beverages. This breakthrough advances the assessment of aromatic and sensory characteristics and underscores the potential for technology-driven improvements in quality control within the industry. González-Viejo et al. [6] further reinforced the significance of machine learning in food assessment, particularly in beer evaluation, by integrating electronic noses with sophisticated algorithms, achieving a new level of precision in flavor profiling. These collective efforts testify to the transformative power of advanced technologies in revolutionizing the landscape of food and beverage characterization.

A diverse array of mathematical models has been observed within machine learning. Principal component analysis (PCA) has been identified as the prevailing analytical tool in this field. PCA effectively visualizes the intensity of various descriptors associated with the analyzed beverage through radar or spider charts [5,6]. Moreover, a notable aspect of this research landscape is the limited diversity of studies investigating the correlation between beverages and their descriptors, encompassing flavor, aroma, quality, palatability, and body [8,14,15]. Many of these studies have been conducted by a select group of researchers, offering valuable insights into the subject [6,16,17]. Within the context of distilled beverages, a conspicuous gap in the research emerges, with most studies predominantly centered on the correlation between fermented beverages like wine and beer [1,6,14]. This presents a substantial area for exploration and investigation, focusing on the correlation of varied distilled beverages such as baijiu, cachaça, gin, mezcal, rum, tequila, and whisk(e)y. These beverages represent a broad spectrum of sensory characteristics and production processes. Each beverage category has unique sensory descriptors due to its raw materials, fermentation, distillation, and aging processes. For instance, whisk(e)y exhibits smoky and roasted profiles, tequila emphasizes citrusy and vegetal notes, and baijiu provides distinct fermented aromas. This diversity offers a robust basis for constructing a comprehensive flavor wheel. The beverage categories were chosen based on global prominence and distinct sensory profiles. However, this selection does not encompass all distilled spirits, and certain cultural or regional beverages might exhibit unique descriptors not reflected in this study.

Understanding the relationship between chemical compounds and sensory descriptors in distilled spirits is critical for advancing quality control, product development, and consumer appreciation. Existing studies have largely focused on qualitative sensory analyses or targeted specific compound–descriptor relationships. However, these approaches lack the scalability and systematic precision needed to address the complexities of modern beverage profiling. This study introduces a novel, machine learning-driven approach to flavor wheel development, offering a quantitative and integrative framework. Specifically, this research aims to: (1) identify chemical–sensory relationships across a broad dataset of distilled spirits; (2) employ principal component analysis (PCA) and clustering to categorize descriptors systematically; and (3) construct a comprehensive, machine learning-informed flavor wheel. These contributions mark a significant advancement over traditional descriptive methods by enabling a data-driven understanding of flavor profiles. The work is structured as follows. Section 2 presents the proposed methodology from the database structure until the flavor wheel development. For this purpose, a PCA approach was considered for dimension reduction and explanatory assessment, and a clustering analysis was used to describe the sensory descriptors. Section 3 presents the results obtained from the data manipulation, PCA analysis, and the clustering algorithm used to develop the flavor wheel. Finally, Section 4 includes the concluding remarks.

## 2. Methodology

### 2.1. Database Construction and Preprocessing

A total of 6 collections of chemical components were used to construct a robust foundation for the database. Through careful data tidying, 3051 distinct chemical components were introduced manually to the archive, each linked to a group of descriptors about their flavor attributes, including both smell and taste. Diverse sources facilitated the identification of a broader range of substances and minimized bias in the modeling approaches. The database comprised various chemical components, incorporating multiple classes of organic and inorganic compounds commonly encountered in flavor and fragrance studies. Descriptors related to flavor were carefully selected based on established sensory terminology, covering a range of characteristics such as fruity, floral, woody, spicy, sweet, sour, and bitter, among others. The database was structured by creating a database ID for each chemical compound accompanied by its IUPAC name and its common name; therefore, each component is followed by its corresponding CAS number, descriptors, and the functional group and distilled spirits to which it belongs, as illustrated in Table 1. This table includes the grouping of rum/cachaça and tequila/mezcal, given their shared characteristics in raw materials, production, and sensory profiles. Both rum and cachaça originate from sugarcane. While rum may be produced from molasses, sugarcane juice, or other derivatives, cachaça is exclusively made from fresh juice. Tequila and mezcal are agave-based spirits, with mezcal encompassing tequila as a subset. Tequila is made exclusively from blue agave, while mezcal allows for a broader range of agave species. However, the production process and raw material create substantial overlaps in descriptors like citrusy, vegetal, and smoky. This commonality makes their flavor profiles overlap significantly in sweet, vegetative, and woody descriptors. Other spirits are analyzed individually due to their unique sensory and chemical distinctions, maintaining the scientific rigor of the study.

**Figure 1:**
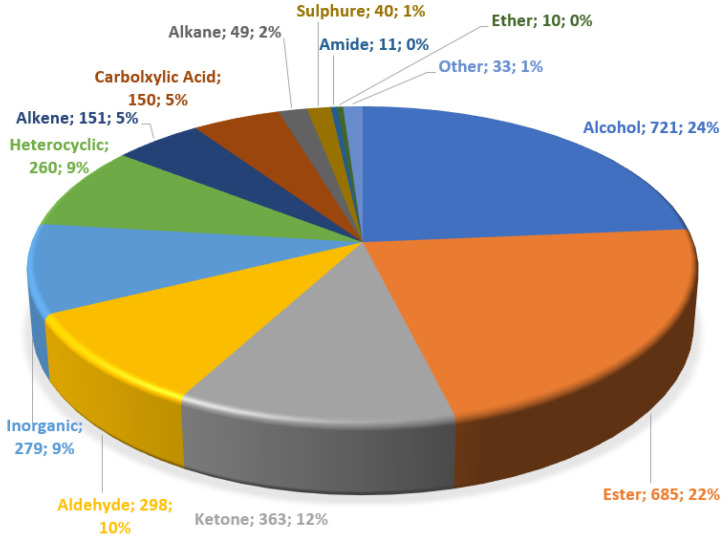
Functional group distribution.

The majority of the database of chemical compounds is composed of about 24% alcohols, 22% esters, 12% ketones, 10% aldehydes, and 9% inorganic compounds. The remaining compounds include heterocyclic compounds, alkenes, carboxylic acids, alkanes, sulphures, amides, and ethers, as shown in Figure 1.

**Figure 2:**
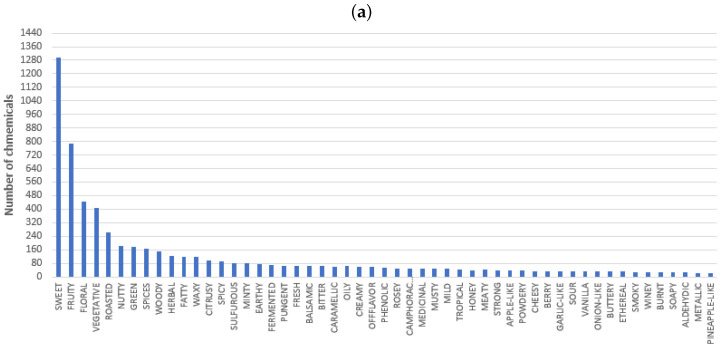

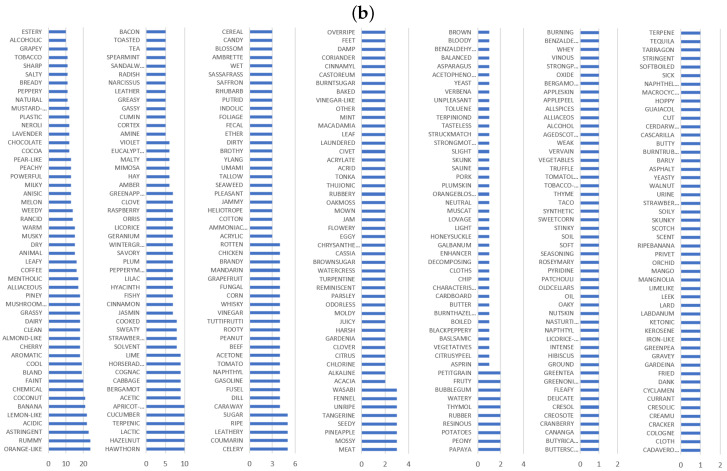
Sensory descriptor distribution of (**a**) 80% and (**b**) 20% of the records.

Regarding the obtained descriptors, Figure 2 depicts the distribution of 80% (53 descriptors) and 20% (350 descriptors) of the records. This figure shows that some of the most common descriptors include sweet, fruity, floral, vegetative, roasted, nutty, green, spices, woody, herbal, fatty, and waxy. On the contrary, some of the most uncommon descriptors include rubber, muscat, green pea, and leek, among others.

The chemical compounds and their descriptors were joined together with the chemicals reported in different references, as those indicated in the book of Hill and Jack [18]. For this purpose, a Visual Basic code was implemented that searched for exact correspondence between vectors of chemical compounds found in different alcoholic beverages and the compounds reported by Hill and Jack [18]. The correspondences were assigned a value of 1, while the remaining values were 0 for each beverage.

**Figure 3:**
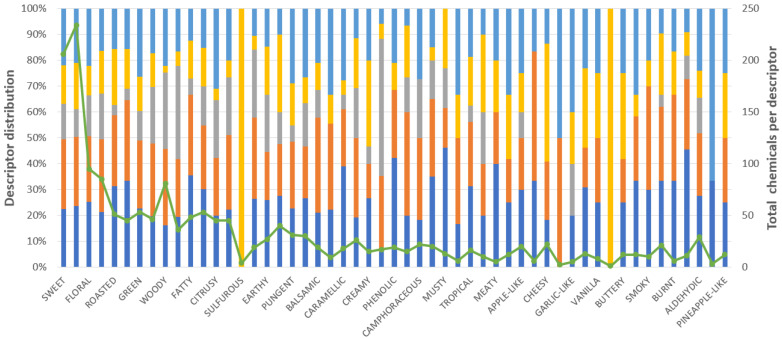
Distribution of descriptors per beverage.

The frequency of each descriptor among the chemical compounds was determined to develop a Pareto analysis that statistically identified the most significant descriptors. The Pareto analysis selected 53 out of 403 descriptors. Figure 3 displays the distribution of the number of chemical compounds from these 53 descriptors in the whisk(e)y, rum/cachaça, gin, baijiu, and tequila/mezcal. This figure depicts interesting patterns. Baijiu is the only beverage reporting sulfurous and onion-like descriptors. Whisk(e)y has, in higher proportion, descriptors such as roasted, nutty, fatty, waxy, earthy, caramelly, phenolic, musty, tropical, meaty, apple-like, burnt, soapy, and aldehydic. Tequila reported descriptors including citrusy, pungent, garlic-like, and metallic. Rum is recognized to have some of the following descriptors: vegetative, green, spices, woody, spicy, minty, balsamic, bitter, oily, rosey, berry, and smoky. Finally, gin has more chemicals associated with herbal descriptors.

As shown in Figure 2, sweet and fruity are the two predominant descriptors in all five beverages (about 25% of the counting). Therefore, it was decided to exclude these descriptors to minimize any bias. The new Pareto analysis selected 74 out of 403 descriptors in this direction. Simultaneously, the frequency of each descriptor was analyzed according to each distilled beverage, showing the tendencies associated with the spirits. This dataset of the counting of chemical compounds in each of these 74 descriptors, i.e., a database of 74 × 5, is implemented in the remainder of the paper. Note that counting the chemicals in this way prevents any problem with missing descriptors in the chemical compounds. Also, using 80% of the data ensures that only the more representative descriptors are implemented, reducing the probability of any relevant inconsistency in the final data.

Normalizing the information was imperative for a more comprehensive data analysis using machine learning. This normalization process was performed in Excel using the “STANDARDIZE” function. This function utilized the average of each descriptor within the alcoholic beverage and the standard deviation of the entire population as inputs, ensuring precise functionality as follows:

(1) $$x_{std}=\frac{x-\mu_{x}}{\sigma_{x}}$$

where $x_{std}$ is the standardized value, *x* is the chemical count obtained by pairing each beverage and the chemical dataset, $\mu_{x}$ is the mean value per beverage, and $\sigma_{x}$ is the standard deviation per beverage.

### 2.2. Exploratory Data Analysis

The previously described database set the basis of the proposed approach. This database contained the main descriptors reported from whisk(e)y, rum/cachaça, gins, baijiu, and tequila/mezcal. The main objective was to extract relevant information using the transpose matrix (5 × 74 matrix). For this purpose, an initial correlation matrix was developed to identify potential relationships and dependencies between the selected descriptors. In addition, the main descriptors were identified for each beverage, pinpointing the top 10–11 descriptors for more in-depth analysis through radar diagrams. Once these descriptors were identified, radar charts were developed for each alcoholic beverage to discern differences between the selected aromas and assess their prevalence. This process also allowed for a comparison with the existing literature to determine the accuracy of the information used.

In addition to the correlation and radar diagrams, a principal component analysis was contemplated to reduce the descriptors’ dimensionality. This enabled a broader comprehension of the fundamental elements influencing the diversity in the chemical setup within the distilled beverages by not only reducing the dimensionality of the data but also identifying the descriptors that contribute the most to the variation in the dataset. The PCA outcomes suggested that specific characteristics were pivotal in shaping the overall chemical characteristics of the spirits and showed which descriptors were positively or negatively correlated with each other.

This analysis was developed through a seven-stage process. The first stage focused on calculating a correlation matrix by computing the pairwise correlations among the dataset descriptors. In the second stage, matrix decomposition techniques were applied to the correlation matrix to extract its eigenvectors and eigenvalues. Eigenvectors signify the directions of maximum variance between the data, while the eigenvalues indicate the magnitude of the variance along these directions.

The subsequent stage involved deciding on the number of principal components based on the ordered eigenvalues, with a typical goal of capturing a significant percentage of the total variance. It was noted that with two principal components, only 67% of the total variance was covered, but with three main components, 88% of the total variance was captured. Following this decision, the original data were transformed into a new principal component space using the selected eigenvectors. Next, using the eigenvectors, a reduced-dimensional matrix was created to present the original dataset in a lower-dimensional space. Finally, this new matrix was visualized into 2D low-dimensional space, showing the relationship between the variables. It is worth mentioning that the statistical methods used in this study, including PCA and clustering, identify patterns within the dataset. However, these methods cannot eliminate the risk of spurious correlations, particularly when dealing with a large and complex dataset.

### 2.3. Clustering Analysis

The exploratory analysis allows a better understanding of the dataset from the beverage point of view, i.e., where the descriptors are related to the variables and the beverages as the registers. However, the main goal of this work is to support the construction of a flavor wheel, where the descriptors are taken as records that need to be related. In this regard, a clustering approach was proposed to associate these descriptors, given the results obtained from the five beverages (i.e., the 74 × 5 matrix is implemented). Many approaches can be used for this clustering; however, this work concentrates on one of the most commonly known: k-means. This method is traditionally implemented as a partition method, where the individuals are divided into K clusters in an iterative relocation process using centroids.

The k-means clustering algorithm is a widely employed and effective unsupervised learning method due to its simplicity, computational efficiency, and scalability in handling large datasets [19]. The algorithm comprises four phases: initialization, assignment, centroid update, and reassignment. In the initialization phase, the algorithm utilizes the elbow plot to determine the optimal number of clusters (K) and randomly assigns each data point to one of the K clusters. Subsequently, the distance between each cluster centroid and every data point is calculated, and each data point is assigned to the nearest centroid. During the centroid update phase, the centroids are recalculated based on the newly appointed data points. Finally, the reassignment process is repeated until the algorithm converges [20].

This method requires defining a metric distance, usually the Euclidean, and determining the necessary number of centroids (i.e., K). The number of centroids can be determined using direct traditional approaches such as the elbow or silhouette methods. The elbow method starts setting the number of centroids as 2, incrementally increasing K by one until reaching the specified maximum value, aligned with the number of variables in the dataset [20,21]. This approach discerns the suitable number of clusters by plotting the within-cluster sum of squares (WCSS) against the iterative values of K, identifying the point at which the graph exhibits a noticeable inflection or “elbow”, denoting a significant reduction. Unfortunately, a notable limitation of this method arises concerning the representation of the elbow point, as it is not consistently explicit in the curve, requiring an artificial determination for cluster number selection [19,20].

The silhouette approach is used to determine the optimal number of clusters in a dataset by measuring how similar an object is to its cluster compared to other clusters. It calculates the silhouette score for each data point, which ranges from −1 to +1. A score close to +1 indicates that the data point is well clustered, while a score near 0 suggests that the point lies between two clusters, and a negative score indicates that the point may be misclassified. By averaging the silhouette scores across all data points for different clusters, one can identify the number of clusters that maximize the average silhouette score, indicating the best-defined clusters [22].

Both methods were applied to determine the number of clusters most effectively describing the relational dynamics among the dataset descriptors. Initially, the raw data underwent transposition. After this transformation, the within-cluster sum of squares was graphically represented utilizing a loop and the *kmeans* (stats package) function in R-Project. The silhouette was determined using an iterative procedure using the function *silhouette* (cluster package). Through this visual analysis, the juncture at which the WCSS commenced a plateauing trend was discerned and subsequently chosen as the number of clusters that best represented the dataset. An alternative function for both cases is *fviz_nbclust* of the factoextra package.

Employing the *kmeans* function and the previously determined number of clusters, the clustering vector was generated and visualized in a two-dimensional (2D) plot using the function *fviz_cluster* with a convex hull. This process involved identifying the centroid associated with each defined cluster and assigning the remaining data points to the nearest cluster centroid, implying that a shorter distance between points indicates a stronger relationship. Subsequently, utilizing the principal components obtained from the principal component analysis (PCA) as the data frame in the first two components, the clusters were assigned and visualized by the arrangement determined by the k-means algorithm [19,20]. Note that these two components should not be confused with those previously mentioned in the exploratory analysis as the dataset uses as registers each descriptor and as a feature the five beverages, i.e., the 75 × 5 matrix.

While this study provides a comprehensive analysis of the selected distilled spirits, the generalizability of the flavor wheel is inherently limited by the scope of beverage categories analyzed. Future research should incorporate a broader spectrum of distilled and fermented beverages to enhance the flavor wheel’s representativeness and ensure its applicability across diverse cultural and production contexts.

### 2.4. Flavor Wheel Approach

After applying the previously mentioned stages to the original data, the resulting clusters were analyzed to identify patterns and relationships among the flavor descriptors. Then, the actual variables were categorized into flavor groups based on their similarities and proximities within the clusters. This categorization helped establish a hierarchical structure of flavor attributes, which ultimately informed the design of the flavor wheel. Simultaneously, those flavor categories were associated based on the chemical compounds present in the different distilled spirits. Next, they were used to create a comprehensive flavor wheel.

The flavor wheel, a critical outcome of this process, was meticulously crafted to visually represent the hierarchical relationships and dependencies among the identified flavor groups. Each sector of the wheel corresponded to a specific flavor category, creating a comprehensive and visually intuitive representation of the interplay between different flavor attributes. Simultaneously, the flavor wheel was distributed in 2 levels: the first level was associated with the principal and dominant characteristics of the components analyzed, and the second one was related to the connections between the descriptors and secondary flavors and aromas. The XLSTAT tool was employed to facilitate the presentation of this intricate dataset and its associations. XLSTAT provided a robust platform for visualizing and interpreting the categorized dataset, offering insights into the interconnections among flavor attributes and their chemical foundations.

## 3. Results and Discussion

### 3.1. Exploratory Analysis

The exploratory analysis is composed of three main analyses. The first is associated with the radar diagrams obtained from the main frequent descriptors from each beverage, followed by a correlation matrix seeking to determine relations among the descriptors, and finally a PCA analysis for describing both these five distilled spirits and the descriptors themselves.

#### 3.1.1. Radar Diagrams

**Figure 4:**
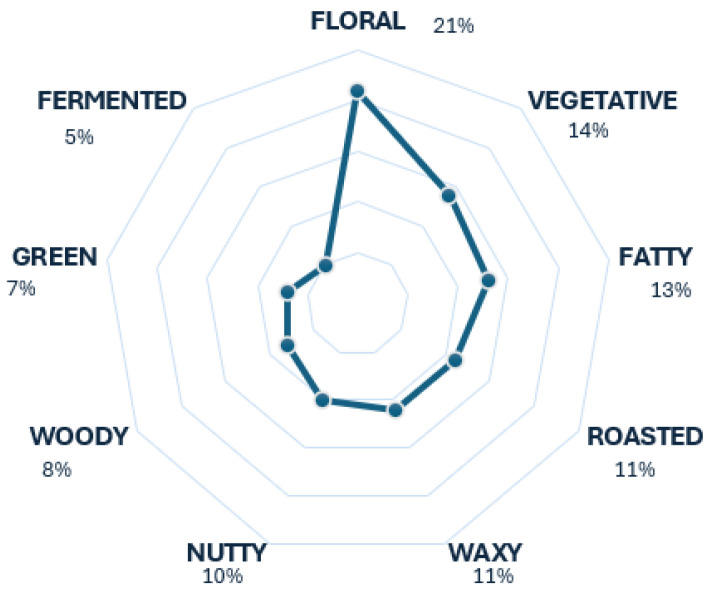
Whisk(e)y’s radar chart.

Figure 4 depicts the results of the whisk(e)y scent profile using the descriptors that were more pronounced in the heat map. This figure indicates that the floral descriptor stands out as the most prevalent among the 10. The accuracy of the chart’s results lies in the fact that the descriptor floral is typically featured in aroma wheels of whisk(e)y as a tier 1 descriptor [18], signifying its prominence. Regarding the other scents, it is accurate to assert that they exhibit good behavior, as these descriptors contribute to the overall aroma of whisk(e)y but less significantly.

**Figure 5:**
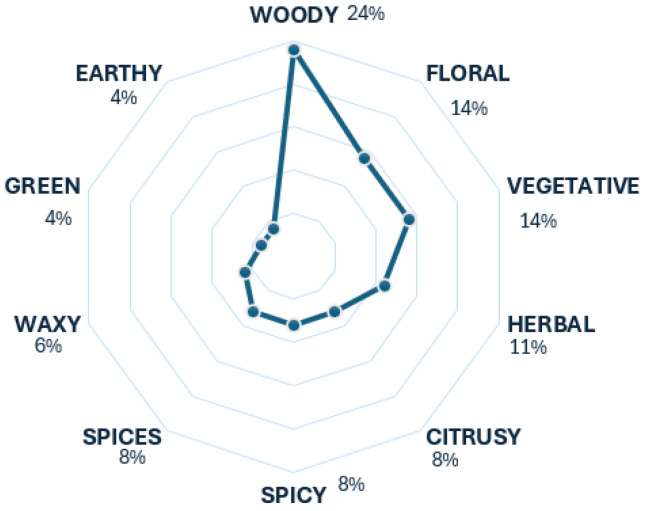
Gin’s radar chart.

In the case of the gin, ten descriptors prevailed, with the woody descriptor being the most predominant, as illustrated in Figure 5. This tendency is correct, as the woody aroma classifies as a tier 1 descriptor for gin. Additionally, there is a slight tendency towards the descriptors floral and vegetative, which is accurate, considering these two aroma scents are also classified as tier 1 descriptors [18]. The lower tendencies observed for the remaining descriptors are correct, as they contribute to the aroma scent of gin to a lesser extent.

**Figure 6:**
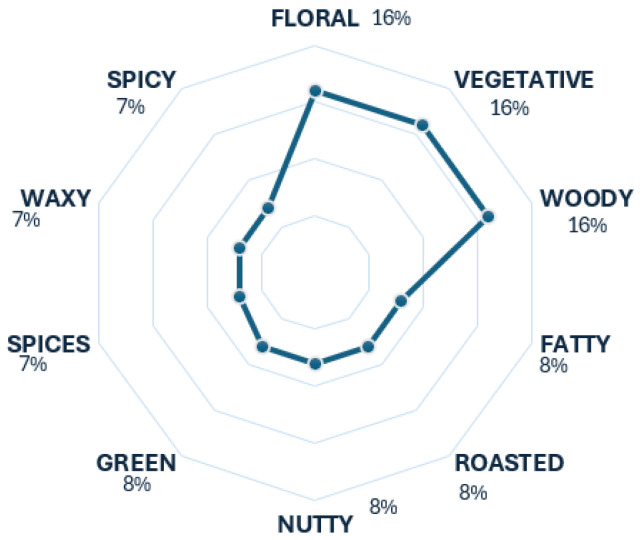
Rum and cachaça’s radar chart.

Rum and cachaça are two spirit beverages made from sugarcane. The critical difference between them is that cachaça has a denomination of origin from Brazil and is made from the juice of sugarcane; meanwhile, rum does not have that denomination of origin, and it can be made from molasses, honey, and the juice of sugarcane. In this case, there is a tendency towards the descriptors floral, vegetative, and woody for these beverages, as seen in Figure 6. This tendency is accurate, considering these two beverages are being considered together, sharing these three aroma scents as tier 1 descriptors [18]. The rest of the descriptors contribute to the scent of rum and cachaça.

**Figure 7:**
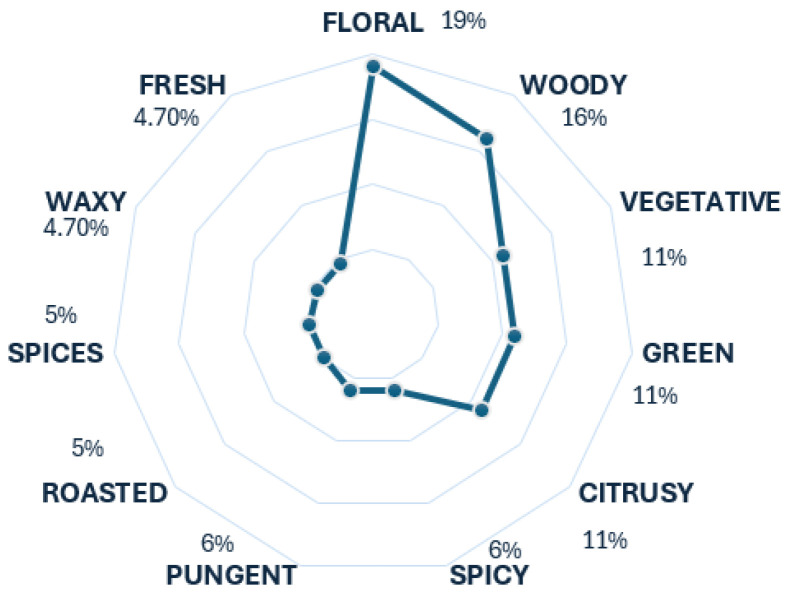
Tequila and mezcal’s radar chart.

Tequila and mezcal are two spirit beverages made from agave plants. The difference is that mezcal can be produced from any agave plant, while tequila can only be made from one specific type: the blue agave. However, considering that tequila is a type of mezcal, it is correct to relate them in terms of their aroma scents. In this case, there is a tendency towards floral and woody descriptors, as seen in Figure 7, considered tier 1 descriptors [18]. These results align accurately with the aromas of these two beverages. The descriptors green and vegetative may appear similar due to their association with vegetative aromas, but there is a distinction between them. ’Green’ typically refers to freshness and greenery, while ’vegetative’ refers to aromas reminiscent of cooked or raw vegetables. Another descriptor usually associated with mezcals is their smokiness, originating from the phenols in the Maillard reaction [18]. Smoky was used in the data, but it did not come out as one of the most popular descriptors for this beverage, which is why it is not in the radar chart. The remaining descriptors contribute to the aroma profiles of tequila and mezcal.

**Figure 8:**
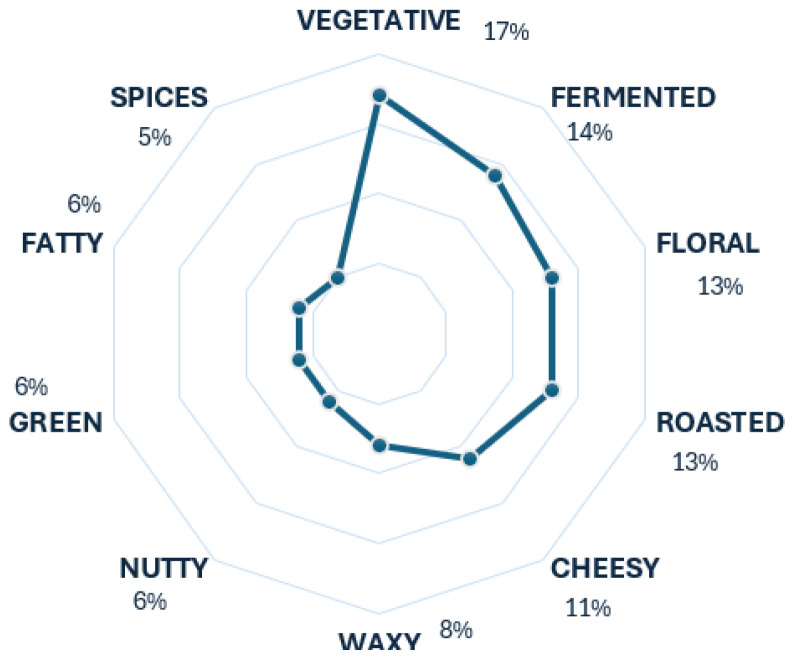
Baijiu’s radar chart.

Baijiu is a typical Chinese spirit beverage made from sorghum. The results provided by the radar chart in Figure 8 show a tendency towards the descriptors vegetative and fermented. This tendency is accurate for the most part, as the fermented descriptor is usually considered a tier 1 element in baijiu aroma wheels [18,23], while vegetative per se is not. However, it can be related to the scent of herbal aroma, another of baijiu’s primary descriptors. The rest of the descriptors are accurate, as they complement the aroma of baijiu as lower tiers.

#### 3.1.2. Correlation Matrix Results

**Figure 9:**
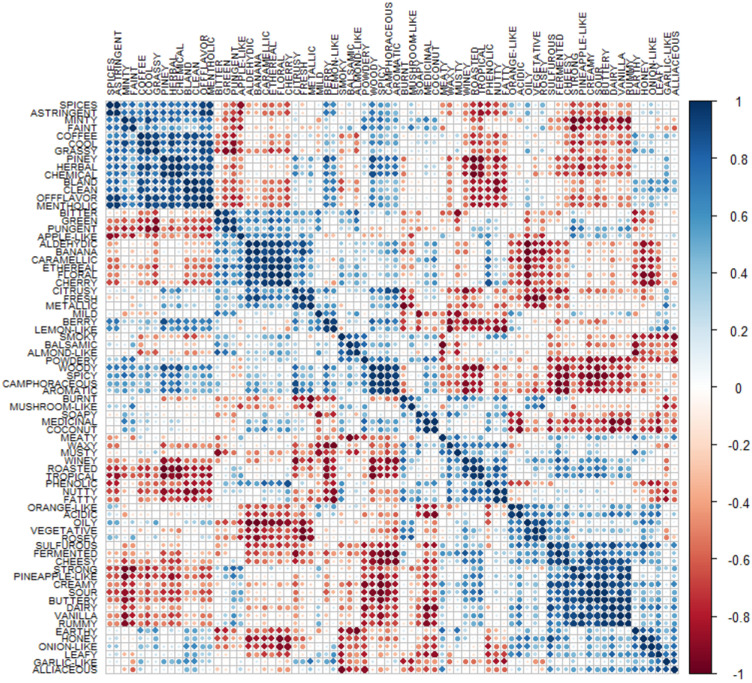
Distilled beverage descriptor correlation matrix.

The correlation matrix presented in Figure 9 shows insightful relationships between the descriptors present in the database. The analysis revealed positive (blue), neutral (white), and negative (red) correlations, indicating the directions of associations between the variables. For instance, blue markers represent a strong positive relation between the variables, meaning that if one is strongly present in a beverage, the other would be too [24,25]. This correlation matrix was determined using the *corr* function in R. using a hierarchical clustering order.

This figure presents interesting results, with highly related descriptors in four main groups. The first one, located in the upper left corner, includes the following descriptors that can be cataloged as “cool scent”: spices, astringent, minty, faint, coffee, cool, grassy, piney, herbal, chemical, bland, clean, and menthol. The second highly correlated group is the one coming from a fruit-based flavors: apple-like, aldehydic, banana, caramel, ethereal, floral, cherry, and citrusy. The third group is more related to the spicy flavor: woody, spicy, aromatic, burnt, and camphoraceous. Finally, the last group is associated with more milky products: vanilla, buttery, creamy, cheesy, rummy, sour, strong, sulfurous, or fermented. Surprisingly, this last group also included the pineapple-like descriptor, which would be more expected in the second cluster mentioned above. These robust clusters are composed of variables with a positive relation. Still, if the correlation is not refined, the clusters include much noise, meaning some relations are not linear [26].

The relations associated with red makers show a strong negative linear relationship between the two variables. In the context of statistical analysis, particularly correlation, a negative correlation means that as one variable increases, the other variable decreases. For example, the descriptor piney in a distilled spirit must guarantee the absence of waxy nuances. Other examples include minty, astringent, or faint with the scents found in the last cluster (e.g., vanilla, buttery, creamy). It is also obtained that the first group (e.g., spices, cool, grassy) is strongly negatively correlated with flavors such as roasted, tropical, phenolic, nutty, fatty, strong, and creamy.

The correlation matrix shows moderated relationships, both negative and positive. A positive correlation of 0.5, associated with the white markers, suggests that as one variable increases, the other variable tends to grow, but not necessarily ideally. A negative correlation of −0.5 shows that as one variable increases, the other tends to decrease but is not perfectly linear [25,27]. Whether a moderate correlation is considered significant is not a precise way to categorize or discard the presence or absence of two variables and their clustering. It is important to emphasize that this representation is practical for superficially understanding the relationship between two specific variables rather than precisely clustering a group of variables.

#### 3.1.3. PCA Results

**Figure 10:**
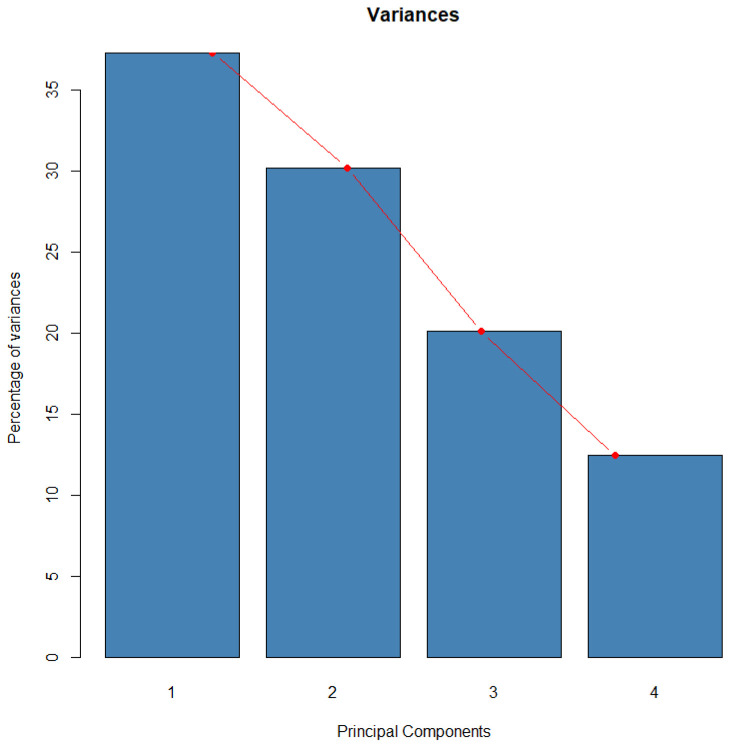
Scree plot to determine the number of PCA components.

The inertia of the initial dimensions indicates the strength of relationships between variables and suggests how many dimensions should be examined. The first two dimensions account for 67.47% of the total dataset inertia, meaning that the plane captures this percentage of the total variability of individuals (or variables), as depicted in the scree plot in Figure 10.

This relatively high percentage suggests that the first plane effectively represents the data variability. Furthermore, this value exceeds the reference threshold of 62.46%, indicating that the variability explained by this plane is significant. The reference value corresponds to the 0.95 quantile of the inertia percentage distribution, derived from simulating 1029 data tables of comparable size based on a normal distribution. This information was obtained using the FactoShinny package in R-Project.

**Figure 11:**
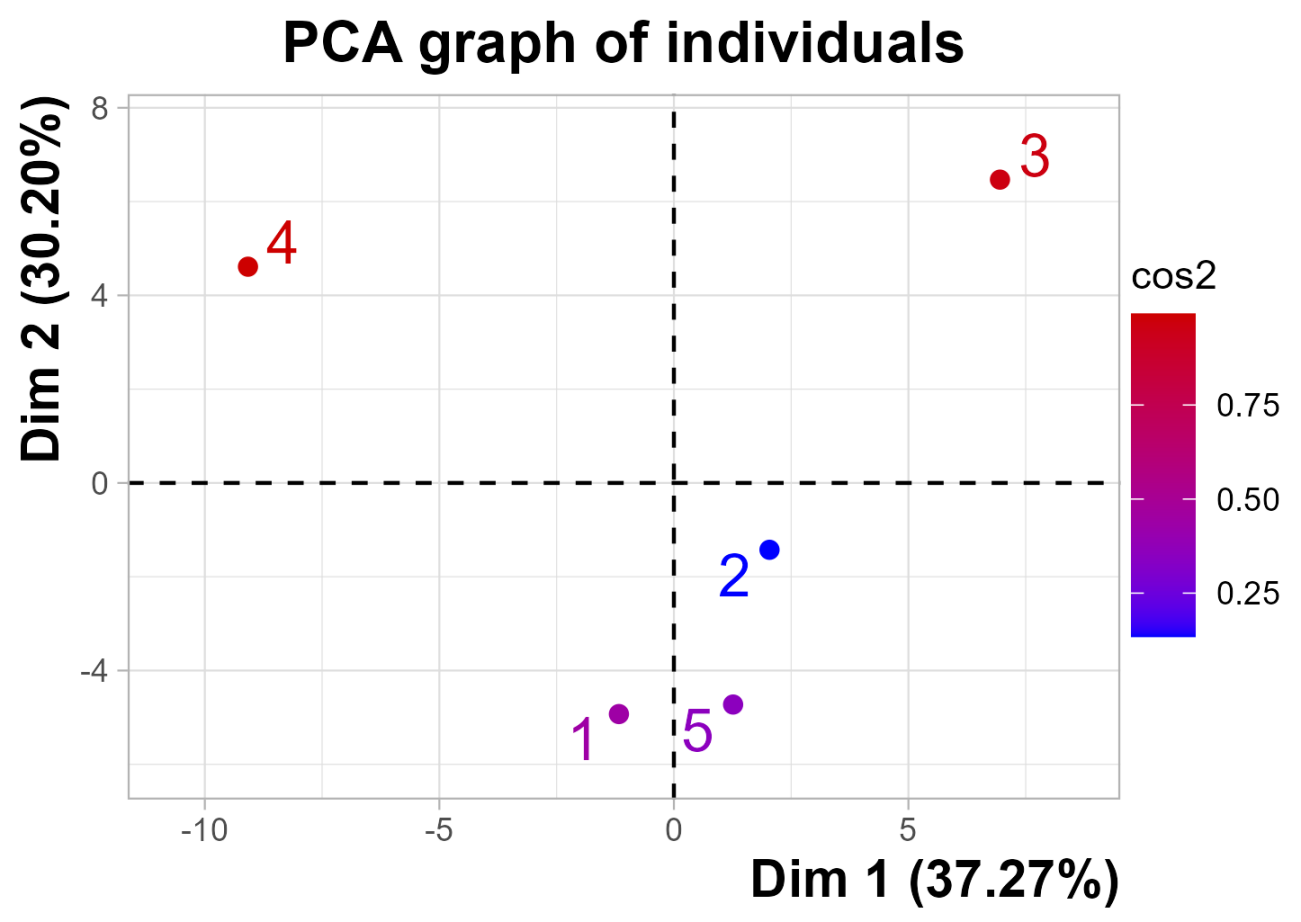
Individual factor map.

**Figure 12:**
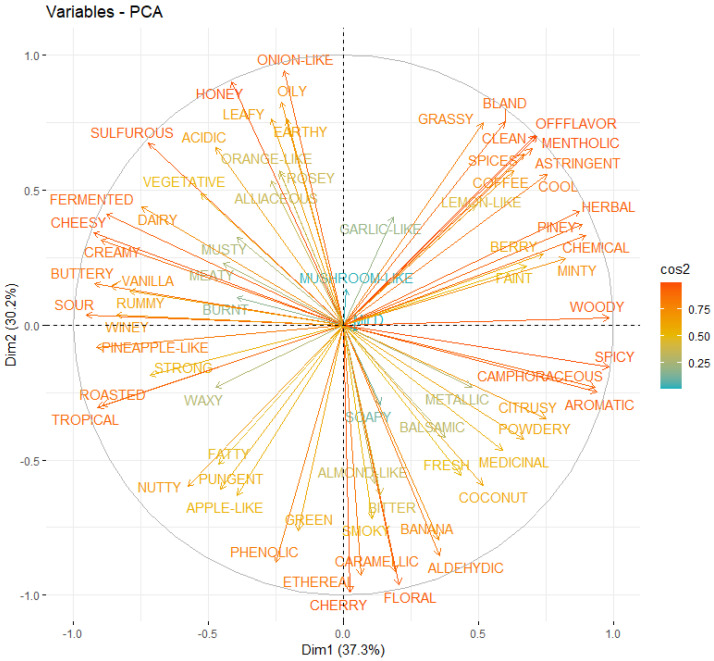
Scree plot to determine the number of PCA components.

In terms of the components, Figure 11 and Figure 12 show the individual and variable factor maps using the PCA for the first two components. Based on these results, it is obtained that the first dimension is separated with a positive value by the third individual (GIN) and the fourth individual (BAIJU) with a strong negative value. These results can be related to those of the descriptor factors and are summarized in Table 2. According to this table, descriptors strongly correlated with GIN are woody, spicy, aromatic, piney, or camphoraceous. Descriptors that are commonly associated with flavors of gins. On the contrary, strong negative values are sour, cheesy, buttery, tropical, and creamy. These results match the strong correlation of previous authors, see Qiao et al. [28], especially those from pineapple, cheesy, and rancid.

The second component distinguishes individuals, such as 1 (whisk(e)y) and 5 (tequila/mezcal), from the upper side of baijiu and gin. Whisk(e)y and tequila/mezcal are characterized as having low values for the variable onion-like. At the same time, as expected, floral and cherry are highly correlated with these beverages, as depicted in Table 3.

These descriptors allow a better understanding of the descriptors from these beverages and how they are correlated based on their number of shared chemicals. However, this PCA was contemplated as an exploratory analysis, bearing in mind that only five registers (associated with each beverage) with 74 features were considered. Indeed, this is a highly challenging problem because tests such as those reported by Barllet may indicate more registers are required, and redundant variables may be reduced using those more correlated variables. While the observed correlations between flavor descriptors and chemical compounds provide valuable insights, they should be interpreted cautiously. The statistical methods employed, such as PCA and clustering, aim to highlight meaningful relationships; however, some correlations may arise coincidentally due to the high dimensionality and complexity of the dataset. Future research incorporating experimental validation, such as controlled sensory evaluations and targeted chemical analyses, is necessary to substantiate these findings.

### 3.2. Clustering

**Figure 13:**
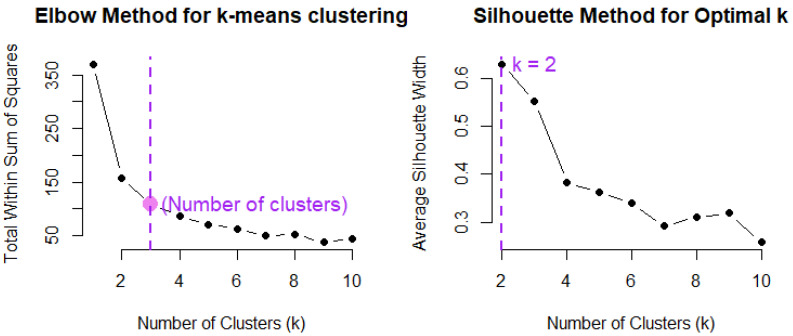
Elbow method (**left**) and Silhouette (**right**) method’s results using k-means clustering.

**Figure 14:**
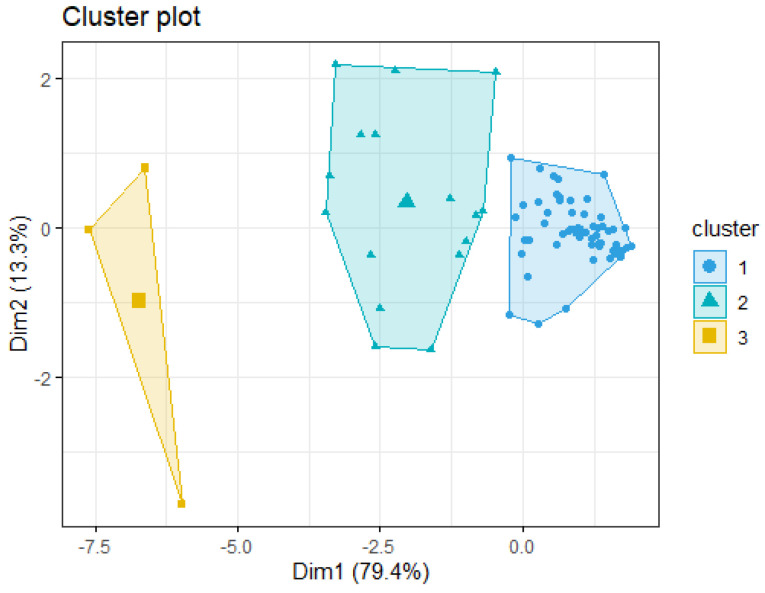
K-means clustering groups of the projected data using the first two PCA components. Note: Clusters match those in Table 4.

The application of the k-means algorithm revealed three clusters by the elbow method and two clusters by the Silhouette method (Figure 13). Considering three clusters, the first comprised 55 variables, the second included 16, and the third included 3. In the case where only two clusters are contemplated, the three points of the third cluster are joined to those in the second one. Table 4 presents the variables included in every cluster and Figure 14 depicts the clusters projected into their first two PCA components. Considering the distance between the second and third clusters, and given the fact that these components covered more than 92% of the variance, this configuration was chosen despite the suggested results from the Silhouette method. Cluster visualization is vital in understanding data’s underlying structure and relationships. By providing clear and intuitive representations of the clusters identified by clustering algorithms, these visualizations facilitate a deeper understanding of the data’s complexity and aid in further analysis and interpretation [29,30]. These clusters provide valuable insights into the underlying structure of the dataset, revealing inherent groupings that can inform further analysis and interpretation [29]. The distribution of variables across the clusters sheds light on the potential heterogeneity within the dataset. This distribution showed that clusters 1 and 2 have a stronger relationship because of the short distance in between. But, between clusters 2 and 1 and cluster 3 there is a clear distinction, indicating distinct differences in the patterns or characteristics of the variables of these clusters.

The insights from the k-means clustering can be applied to sensory evaluation and product development in distilled beverages. Sensory experts can better characterize and differentiate between beverages by understanding descriptors’ relationships and dominance nuances. At the same time, product developers can tailor their creations to specific flavor profiles or consumer preferences. Future research could explore alternative clustering algorithms, consider incorporating additional data sources, and investigate the applicability of these findings to a broader range of distilled beverages.

### 3.3. Flavor Wheel Development

**Figure 15:**
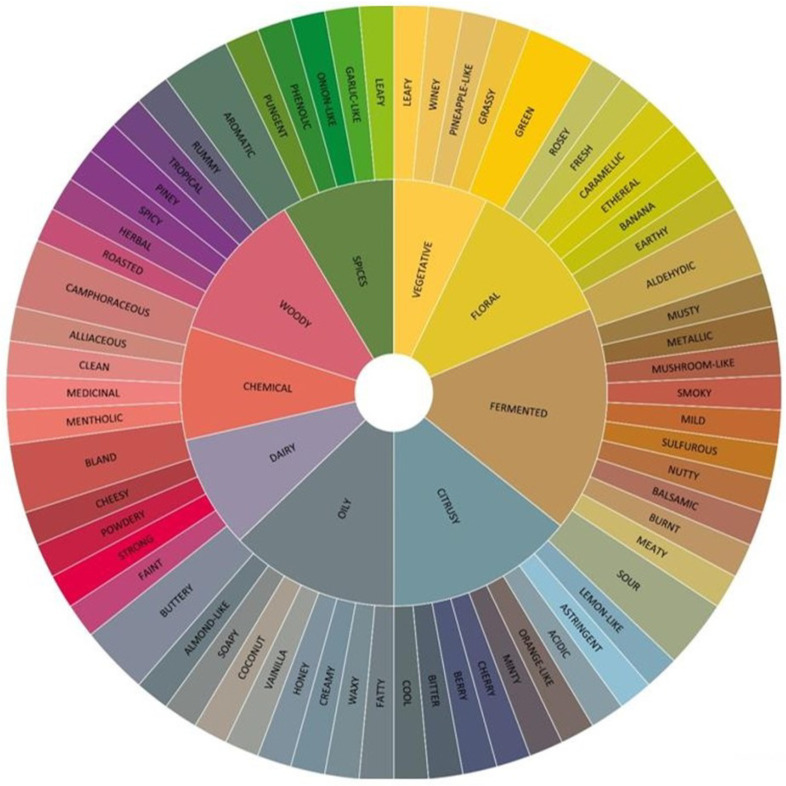
Distilled beverage flavor wheel.

Using the k-means algorithm for clustering and leveraging the correlation matrix, the flavor wheel was created to categorize and inter-relate descriptors independently of the distilled beverage under consideration. Illustrated in Figure 15, the flavor wheel unfolds across two primary levels. The initial tier unveils cluster-based categories organized through correlation-driven hierarchies. This tier is further stratified into nine subsections, each chosen due to its representation of dominant flavors or aromas within the respective clusters [31].

According to this framework, the components of cluster number 3, “VEGETATIVE”, “FLORAL”, and “WOODY”, were selected to be part of the first level because of the frequency of those among the databases. Furthermore, from cluster 2, descriptors such as “CITRUSY”, “FERMENTED”, “SPICES”, and “OILY” were included in the first level according to their closeness to the centroid of the cluster. These descriptors demonstrated robust relationships with many other descriptors, underscoring their centrality within the overarching flavor profile. From the first cluster, the descriptors “DAIRY” and “CHEMICAL” occupy a predominant position within this cluster. On the other hand, the second level of the flavor wheel represents the principal relations between the first-level descriptors and the other 70 descriptors of the database.

As such, the flavor wheel serves as a classification tool and a dynamic visual representation of the intricate relationships and dominance nuances that characterize the diverse and rich flavors and aromas across distilled beverages.

This flavor wheel is a crucial graphical tool in sensory evaluation, specifically in the distilled spirits industry. Additionally, it acts as a guiding instrument during tastings, enhancing the exploration of various sensory elements and fostering a more nuanced appreciation of the subtleties in products like baijiu, cachaça, gin, mezcal, rum, tequila, and whisk(e)y.

This flavor wheel was developed using only a few descriptors, so one limitation lies in its potential need for more exhaustive coverage of the full spectrum of sensory experiences. The limited number of descriptors may lead to a simplification of the sensory landscape. On the other hand, the selected Pareto descriptors used to create the flavor wheel may have been directed toward a specific distilled beverage, introducing a potential source of bias. Suppose the descriptors were primarily derived from the sensory profiles of a particular type of beverage, such as whisk(e)y or gin. In that case, the resulting wheel may not generalize well to other categories. This specificity could limit the applicability and universality of the flavor wheel across a broader range of products.

## 4. Conclusions

The dataset employed and generated for this project has effectively fulfilled its objective by furnishing precise information concerning chemical compounds and their descriptors. It facilitated the classification of diverse chemical compounds into their respective spirit beverages and enabled an in-depth analysis of aromas that may find application in future endeavors. This study successfully met its stated objectives. PCA and correlation analysis identified the relationships between chemical compounds and sensory descriptors, providing a foundational understanding of flavor profiling in distilled spirits. A comprehensive two-level flavor wheel was developed, offering a practical tool for distillers, sensory experts, and consumers to interpret and apply these findings. Additionally, using machine learning techniques such as PCA and k-means clustering effectively enhanced the analysis, enabling a data-driven approach to flavor characterization.

However, for the seamless continuation of this project, it is imperative to enhance the dataset. While the existing outcomes are accurate, they exhibit a degree of generality. To attain more refined and comprehensive results, it is necessary to categorize the descriptors into distinct tiers. This categorization will augment the correlation with their corresponding beverages and enrich the exploration of their aromas, rendering the study more comprehensive. While this work identified robust relationships within the selected distilled spirits, future research should extend these findings to a broader range of beverages to enhance generalizability and refine the flavor wheel’s applicability.

The results obtained from the radar charts have been instrumental in the positive trajectory of the project. These initial findings contribute to analyzing aromas in the various spirit beverages examined in this study. Subsequently, these findings are juxtaposed with diverse aroma wheels associated with each beverage. This comparative analysis illuminates the disparities between the obtained results and those in the experimental domain.

On the other hand, the correlation matrix analysis revealed strong and moderate relationships between the descriptors in the beverage database. Strong relationships suggest a clear association between the variables, with one variable’s presence implying the presence of the other. Moderate relationships indicate a less defined association, where an increase in one variable tends to correspond to an increase or decrease in the other variable, but not necessarily in a perfectly linear manner. The analysis also identified negative linear relationships between variables. These relationships suggest that the other variable decreases consistently as one variable increases. Although the correlation matrix provides essential information for data analysis and the subsequent creation of the flavor wheel, it also includes general information about the relationships between descriptors. It needs to be more precise to determine whether two descriptors will always be present in a particular beverage, regardless of the type of distillate.

The PCA findings not only corroborated aspects of the correlation matrix but also extended our comprehension of the intricate relationships among the variables in a more spatially contextualized manner. This dimensional reduction allowed for a more condensed and interpretable representation of the data, facilitating the identification of trends and correlations among variables. The 3D PCA offered a more detailed view of complex relationships, while the 2D PCA provided a simplified representation for more straightforward interpretation. The spatial arrangement of variables highlighted relationships’ strength and directionality, offering a holistic view of how descriptors coalesced in distinct plot regions.

Applying the k-means algorithm effectively partitioned the dataset into three clusters, each comprising a distinct set of variables. The first cluster, with 55 variables, represented the largest group, while the second and third clusters, with 16 and 3 variables, respectively, represented smaller but still significant subsets of the data. The formation of these clusters provided valuable insights into the underlying structure of the dataset, suggesting that the variables were not randomly distributed but rather exhibited inherent groupings. The k-means clustering results complemented the findings of the PCA by providing a more concrete and granular understanding of the variable groupings within the dataset. While PCA effectively identified the overall patterns and relationships within the data, k-means clustering allowed for the identification of specific clusters of variables that share common characteristics.

Finally, the flavor wheel, created using the k-means algorithm and the correlation matrix, is a valuable tool for categorizing and interrelating descriptors independent of the distilled beverage under consideration. This visual representation effectively captures the intricate relationships and dominance nuances that characterize the diverse and rich flavors and aromas across distilled beverages. One limitation of the flavor wheel is its potential lack of exhaustive coverage of the full spectrum of sensory experiences. The limited number of descriptors used may lead to a simplification of the sensory landscape. This specificity could limit the applicability and universality of the flavor wheel across a broader range of beverages. Despite its limitations, it is a valuable reference for tasters, evaluators, and product developers. It facilitates a deeper understanding of the intricate relationships and dominant nuances that characterize these beverages’ diverse and rich flavors and aromas.

While this study provides a comprehensive analysis of selected distilled spirits, the generalizability of the flavor wheel is inherently limited by the scope of the beverage categories analyzed. Future research should incorporate a broader spectrum of distilled and fermented beverages to enhance the representativeness of the flavor wheel and ensure its applicability across diverse cultural and production contexts. Subsequent studies should consider incorporating data from additional beverage categories such as vodka, brandy, and traditional spirits from diverse regions to enrich the dataset and enhance the applicability of the flavor wheel. In addition, future research should aim to validate the observed correlations through experimental methods, such as controlled addition or removal of compounds in sensory tests, to distinguish genuine relationships from coincidental associations.

## Figures and Tables

**Table 1:** Database structure.

ID	Common Name	IUPAC Name	CAS	Descriptors	Functional Group	W	R/C	G	B	T/M
1	1-Aminopropan-2-Ol	1-aminopropan-2-ol	78-96-6	FISHY	Alcohol	0	0	0	0	0
2	3-Methyl-2-Oxobutanoic-Acid	3-methyl-2-oxobutanoic acid	759-05-7	FRUITY	Carboxylic Acid	0	0	0	0	0
3	2-Oxobutanoic-Acid	2-oxobutanoic acid	600-18-0	CREAMY/SWEET/ROASTED/NUTTY/CARAMELLIC/LACTIC	Carboxylic Acid	0	0	0	0	0
⋮				⋮						⋮
3050	Geosmin	4a(2H)-naftalenol,octahidro-4,8a-dimetil-, (4 S, 4a S, 8a R)-	19700-21-1	SULPHUROUS/ ROASTED/MEAT	Inorganic	0	0	0	1	0

W: whisk(e)y, R/C: rum/cachaça, G: gin, B: baijiu, T/M: tequila/mezcal

**Table 2:** Descriptors highly correlated with the first component.

Descriptor	Correlation	*p*-Value
Woody	0.98	2.73 $\times \;10^{-3}$
Spicy	0.98	2.93 $\times \;10^{-3}$
Aromatic	0.94	1.85 $\times \;10^{-2}$
Camphoraceous	0.93	2.12 $\times \;10^{-2}$
Chemical	0.90	3.91 $\times \;10^{-2}$
Piney	0.88	4.67 $\times \;10^{-2}$
Roasted	−0.90	4.02 $\times \;10^{-2}$
Creamy	−0.90	3.92 $\times \;10^{-2}$
Tropical	−0.91	3.17 $\times \;10^{-2}$
Pineapple-like	−0.91	2.98 $\times \;10^{-2}$
Buttery	−0.92	2.65 $\times \;10^{-2}$
Cheesy	−0.93	2.41 $\times \;10^{-2}$
Sour	−0.95	1.28 $\times \;10^{-2}$

**Table 3:** Descriptors highly correlated with the second component.

Descriptor	Correlation	*p*-Value
Onion-like	0.94	1.69 $\times \;10^{-2}$
Honey	0.90	3.71 $\times \;10^{-2}$
Phenolic	−0.88	5.00 $\times \;10^{-2}$
Caramellic	−0.91	2.97 $\times \;10^{-2}$
Ethereal	−0.93	2.40 $\times \;10^{-2}$
Floral	−0.96	8.88 $\times \;10^{-3}$
Cherry	−0.99	1.43 $\times \;10^{-3}$

**Table 4:** K-means-obtained clusters.

Cluster 1		Cluster 2	Cluster 3
SULFUROUS	RUMMY	ROASTED	FLORAL
MINTY	ASTRINGENT	NUTTY	VEGETATIVE
BALSAMIC	ACIDIC	GREEN	WOODY
BITTER	LEMON-LIKE	SPICES	
CARAMELLIC	BANANA	HERBAL	
CREAMY	COCONUT	FATTY	
OFF FLAVOR	CHEMICAL	WAXY	
PHENOLIC	FAINT	CITRUSY	
ROSEY	BLAND	SPICY	
CAMPHORACEOUS	COOL	EARTHY	
MEDICINAL	AROMATIC	FERMENTED	
MUSTY	CHERRY	PUNGENT	
MILD	ALMOND-LIKE	FRESH	
TROPICAL	CLEAN	OILY	
HONEY	DAIRY	CHEESY	
MEATY	GRASSY	ALDEHYDIC	
STRONG	MUSHROOM-LIKE		
APPLE-LIKE	PINEY		
POWDERY	ALLIACEOUS		
BERRY	MENTHOLIC		
GARLIC-LIKE	COFFEE		
SOUR	LEAFY		
VANILLA	WINEY		
ONION-LIKE	BURNT		
BUTTERY	SOAPY		
ETHEREAL	METALLIC		
SMOKY	PINEAPPLE-LIKE		
ORANGE-LIKE			

## Data Availability

The original contributions presented in this study are included in the article/Supplementary Materials. Further inquiries can be directed to the corresponding authors.

## Supplementary Materials

The database used for this analysis can be downloaded at: https://www.mdpi.com/article/10.3390/foods13244142/s1.

## References

[1] Gonzalez Viejo C., Fuentes S., Godbole A., Widdicombe B., Unnithan R. (2020). Development of a low-cost e-nose to assess aroma profiles: An artificial intelligence application to assess beer quality. Sens. Actuators B Chem..

[2] Voss H., Mendes Júnior J., Farinelli M., Stevan S. (2019). A Prototype to Detect the Alcohol Content of Beers Based on an Electronic Nose. Sensors.

[3] Barnes Q., Vial J., Thiébaut D., De Saint Jores C., Steyer D., Contamin M.A., Papaiconomou N., Fernandez X. (2022). Characterization of Flavor Compounds in Distilled Spirits: Developing a Versatile Analytical Method Suitable for Micro-Distilleries. Foods.

[4] Huang H., Chen Y., Hong J., Chen H., Zhao D., Wu J., Li J., Sun J., Sun X., Huang M. (2024). Unraveling the chemosensory characteristics on different types of spirits based on sensory contours and quantitative targeted flavoromics analysis. Food Chem. X.

[5] Hu Q., Sellers C., Sang-Il Kwon J., Wu H.J. (2022). Integration of surface-enhanced Raman spectroscopy (SERS) and machine learning tools for coffee beverage classification. Digit. Chem. Eng..

[6] Gonzalez Viejo C., Fuentes S., Torrico D., Howell K., Dunshea F. (2018). Assessment of Beer Quality Based on a Robotic Pourer, Computer Vision, and Machine Learning Algorithms Using Commercial Beers. J. Food Sci..

[7] Sampaio A., Dragone G., Vilanova M., Oliveira J., Teixeira J., Mussatto S. (2013). Production, chemical characterization, and sensory profile of a novel spirit elaborated from spent coffee ground. LWT-Food Sci. Technol..

[8] Gonzalez Viejo C., Fuentes S., Torrico D., Howell K., Dunshea F. (2018). Assessment of beer quality based on foamability and chemical composition using computer vision algorithms, near infrared spectroscopy and machine learning algorithms. J. Sci. Food Agric..

[9] Calvert M., Neill C., Ac-Pangan M., Stewart A., Lahne J. (2024). Development of a hard cider flavor wheel using free word sorting and multivariate statistical techniques. J. Sens. Stud..

[10] Hamilton L., Lahne J. (2020). Fast and automated sensory analysis: Using natural language processing for descriptive lexicon development. Food Qual. Prefer..

[11] Wang J.Q., Dai Z.S., Gao Y., Wang F., Chen J.X., Feng Z.H., Yin J.F., Zeng L., Xu Y.Q. (2023). Untargeted metabolomics coupled with chemometrics for flavor analysis of Dahongpao oolong tea beverages under different storage conditions. LWT.

[12] Yu M., Zheng C., Xie Q., Tang Y., Wang Y., Wang B., Song H., Zhou Y., Xu Y., Yang R. (2022). Flavor Wheel Construction and Sensory Profile Description of Human Milk. Nutrients.

[13] Ramirez J., León J., Amaya-Gómez R., Ratkovich N. (2024). Assessing botanical compatibility in gin production: A mathematical model and network analysis approach. Food Bioprod. Process..

[14] Gonzalez Viejo C., Torrico D., Dunshea F., Fuentes S. (2019). Development of Artificial Neural Network Models to Assess Beer Acceptability Based on Sensory Properties Using a Robotic Pourer: A Comparative Model Approach to Achieve an Artificial Intelligence System. Beverages.

[15] Gonzalez Viejo C., Fuentes S., Howell K., Torrico D., Dunshea F. (2018). Robotics and computer vision techniques combined with non-invasive consumer biometrics to assess quality traits from beer foamability using machine learning: A potential for artificial intelligence applications. Food Control.

[16] Gonzalez Viejo C., Fuentes S. (2022). Digital Detection of Olive Oil Rancidity Levels and Aroma Profiles Using Near-Infrared Spectroscopy, a Low-Cost Electronic Nose and Machine Learning Modelling. Chemosensors.

[17] Gonzalez Viejo C., Torrico D., Dunshea F., Fuentes S. (2019). Emerging Technologies Based on Artificial Intelligence to Assess the Quality and Consumer Preference of Beverages. Beverages.

[18] Hill A., Jack F. (2023). Distilled Spirits.

[19] Syakur M., Khotimah B., Rochman E., Satoto B. (2018). Integration K-Means Clustering Method and Elbow Method For Identification of The Best Customer Profile Cluster. IOP Conf. Ser. Mater. Sci. Eng..

[20] Pham D., Dimov S., Nguyen C. (2005). Selection of K in K-means clustering. Proc. Inst. Mech. Eng. Part C J. Mech. Eng. Sci..

[21] Shi C., Wei B., Wei S., Wang W., Liu H., Liu J. (2021). A quantitative discriminant method of elbow point for the optimal number of clusters in clustering algorithm. EURASIP J. Wirel. Commun. Netw..

[22] Rousseeuw P. (1987). Silhouettes: A graphical aid to the interpretation and validation of cluster analysis. J. Comput. Appl. Math..

[23] Li J., Zhang Q., Sun B. (2023). Chinese Baijiu and Whisky: Research Reservoirs for Flavor and Functional Food. Foods.

[24] Lee S.K. (1985). Analysis of covariance and correlation structures. Comput. Stat. Data Anal..

[25] Graffelman J., de Leeuw J. (2023). Improved Approximation and Visualization of the Correlation Matrix. Am. Stat..

[26] Ramasubramanian K., Singh A. (2019). Machine Learning Using R.

[27] Bedeian A.G. (2015). More than Meets the Eye: A Guide to Interpreting the Descriptive Statistics and Correlation Matrices Reported in Management Research. Rev. Ibero-Am. Estratég..

[28] Qiao L., Wang J., Wang R., Zhang N., Zheng F. (2023). A review on flavor of Baijiu and other world-renowned distilled liquors. Food Chem. X.

[29] Ding C., He X. K-means clustering via principal component analysis. Proceedings of the Twenty-First International Conference on Machine Learning.

[30] Al-Omary A.Y., Jamil M.S. (2006). A new approach of clustering based machine-learning algorithm. Knowl.-Based Syst..

[31] De Pelsmaeker S., De Clercq G., Gellynck X., Schouteten J. (2019). Development of a sensory wheel and lexicon for chocolate. Food Res. Int..

